# Toughening and Heat-Resistant Modification of Degradable PLA/PBS-Based Composites by Using Glass Fiber/Silicon Dioxide Hybrid Fillers

**DOI:** 10.3390/polym14163237

**Published:** 2022-08-09

**Authors:** Junchang Gao, Yadong Wu, Jun Li, Xuqiang Peng, Dewu Yin, Jichang Wang, Xiaohua Wang, Meijin Jin, Zengwen Yao, Xiaojun Shen, Shun Wang, Huile Jin

**Affiliations:** 1 Key Laboratory of Leather of Zhejiang Province, College of Chemistry and Materials Engineering, Wenzhou University, Wenzhou 325035, China; 2 Institute of New Materials and Industrial Technology, Wenzhou University, Wenzhou 325035, China; 3 Department of Chemistry and Biochemistry, University of Windsor, Windsor, ON N9B3P4, Canada; 4 Huafon Group Co., Ltd., Kaifaqu Road 1688, Ruian Economic Development Zone, Wenzhou 325299, China

**Keywords:** polylactic acid (PLA), polybutylene glycol succinate (PBS), composites, heat resistance, toughness

## Abstract

In this paper, to enhance the toughness and heat resistance properties of polylactic acid (PLA)/polybutylene succinate (PBS) composites, the PLA/PBS matrix was modified by different glass fiber (GF), GF/SiO_2_, and GF/(Polyaluminium chloride) PAC fillers. Additionally, the effect of filler type, filler content, components interaction and composite structure on the mechanical and thermal properties of the PLA/PBS composites was researched. The results showed that the addition of GF, GF/SiO_2_ and GF/PAC make the PLA/PBS composites appear significantly higher mechanical properties compared with the pristine PLA/PBS composite. Among the different inorganic fillers, the 10%GF/1%SiO_2_ fillers showed excellent strengthening, toughening and heat resistant effects. Compared with the pristine PLA/PBS matrix, the tensile strength, elastic modulus, flexural strength, flexural modulus and Izod impact strength improved by 36.28%, 70.74%, 67.95%, 66.61% and 135.68%, respectively. Considering the above, when the weight loss rate was 50%, the thermal decomposition temperature of the 10%GF/1%SiO_2_ modified PLA/PBS composites was the highest 412.83 °C and its Vicat softening point was up to 116.8 °C. In a word, the 10%GF/1%SiO_2_ reinforced PLA/PBS composites exhibit excellent mechanical and thermal properties, which broadens the application of biodegradable materials in specific scenarios.

## 1. Introduction

With the development of the economy and society, polymer materials play an important role in industrial production, especially in the fields of electronics, chemical industry, and medical treatment. Since the synthesis of artificial plastics in the early 20th century, polymer materials have been applied in different fields and have been fully optimized and developed. At first, chemical polymers produced by the petroleum industry were favored due to their low cost, easy production, good mechanical properties, and excellent heat resistance [1,2,3]. However, due to their rapid production, petroleum-based polymer materials have a significantly negative impact on resources, economy, and environmental security, especially in terms of environmental security, because plastics are not easy to completely degrade spontaneously in the natural environment, especially in the case of the large-scale use of plastic film covers and packaging, resulting in a large amount of waste, causing serious pollution and damage to the ecosystem [4,5,6]. The damage caused by industrial development to the environment and the threat to human health costs millions of dollars to deal with every year. Therefore, the research and development of renewable and biodegradable plastics are attracting more and more attention [7,8,9,10,11,12,13]. Biodegradable materials can gradually degrade spontaneously in the natural environment, and, finally, enter nature in the form of small molecules. As far as biodegradable materials are concerned, polylactic acid (PLA) is widely welcomed because it has good thermoplasticity and is conducive to its preparation and processing [14,15,16]. PLA is lactic acid (LA) derivative produced from renewable resources such as wheat, straw, corn, and sorghum. It is completely biodegradable and can be decomposed into water and carbon dioxide by microorganisms [17,18,19,20]. Due to the global problem of pollution, PLA is considered the most promising biodegradable polymer material in the market, so the application of PLA has become a key step to improving the environment. At present, PLA has been widely used in packaging materials, clothing, and medical devices [21,22,23,24,25]. However, PLA also has obvious defects, such as its brittleness, low toughness, and poor heat resistance when used above its glass transition temperature, so it must be improved to expand its application. The elongation at break of PLA is less than 10% and the impact strength is relatively poor, which limits its application in some special fields [26]. Although its tensile strength and elastic modulus are equivalent to those of polyethylene terephthalate (PET) and polystyrene (PS), the toughness of polylactic acid is poor, which limits its application in plastic deformation requiring a higher stress level [27,28]. For decades, enhancing the toughness of polylactic acid has been widely studied in academic and industrial fields. The mechanical strength of polylactic acid should be enhanced to improve its performance and achieve balanced mechanical properties so that it can be used more widely as a suitable substitute for low-cost traditional petroleum-based polymers. At present, a variety of methods and modification technologies have been developed to enhance the toughness of polylactic acid, including plasticization [29,30], blending [31,32], copolymerization [33], and filler [34,35]. Among them, blending modification is an economical and convenient method to develop new high-performance composites, which combines the advantages of many existing polymers, and the properties of the resulting composites can be adjusted by changing the composition of the mixture. Therefore, many high toughness polymers have been mixed with PLA to improve the toughness of the final composites [36].

At present, Zhou et al. [37] synthesized degradable polyurethane elastomer containing poly (L-lactide) and poly (D-lactide) based on the conclusion that excellent interfacial strength is conducive to the shear yield of unannealed samples, and used it as a toughening modifier for the toughening modification of PLA. The test results show that the impact strength of the composite is more than 50 kJ/m^2^, which is 24 times that of pure PLA. Meanwhile, Petchwattana et al. [38] achieved the purpose of toughening polylactic acid by blending with triglyceride and polymethylmethacrylate co ethyl acrylate, which can improve the impact strength by nearly 23 times based on PLA. In addition, Li et al. [39] prepared a polylactic acid/graphene oxide/acetylated lignin composite film, which increased the elongation at break of PLA by 197%. However, there are rarely reports of using the high toughness polymer PBS to mix with the PLA as the matrix and using different inorganic fillers to synergistically enhance the toughness and heat resistance properties of the PLA/PBS composites.

In this paper, PBS, which was easy to mix with PLA, was selected as the matrix and modified with different inorganic fillers (GF, GF/SiO_2_, and GF/PAC) to study the PLA/PBS composites’ toughness and heat-resistant properties, focusing on the effects of different composite component content, composite component interaction and composite structure on the mechanical and thermal properties of the composites. The tensile and flexural properties was tested by the servo-controlled tensile testing machine, The impact of the properties was tested by the impact test machine. The microstructure of the fracture surface of the different PLA/PBS composites was observed by using a scanning electron microscope. The Vicat softening point (VST) of the sample was tested with a computerized thermal deformation temperature tester. The heat resistant properties of different PLA/PBS composites was characterized using the TG and DSC test.

## 2. Experimental

### 2.1. Materials

PLA (201) was provided by Zhejiang Haizheng biomaterials Co., Ltd. (Taizhou, China). PBS (803S) was purchased from Xinjiang Lanshan Tunhe Company (Changji, China). Food grade talc powder (talc, 1250 mesh) was obtained from Huiteng Chemical Co., Ltd. (Jinan, China). Chain extender (adr4468) was provided by BASF Ag (Ludwigshafen, Germany). Tributyl citrate (TBC, industrial grade) was provided by Sinochem (Jinan, China). Erucic acid amide (ERU, super grade) was provided by Zhilian Plastic Technology Co., Ltd. (Jian, China). Glass fiber (GF, 3 mm, 12 mm) was provided by Taixin Composite Material Co., Ltd. (Zibo, China). Silica (SiO_2_, 5000 mesh) was provided by Tuoyi New Materials Co., Ltd. (Guangzhou, China). Polyaluminium chloride (PAC, gb15892-2013) was provided by Yongping Industrial Raw Materials Co., Ltd. (Bengbu, China).

### 2.2. Fabrication of Samples

Firstly, the PLA and PBS materials were put into the electric heating constant temperature blast dryer and dried at 80 °C for 10 h. Secondly, the mixer was used to evenly mix the dried materials into the basic masterbatch according to a certain proportion (the proportion of each part is PBS:PLA = 63%:27%), and then inorganic substances were added in different proportions on the basic masterbatch according to Table 1. Thirdly, the mixed materials were melt-blended using a parallel co-rotating twin-screw extruder (110 °C~185 °C; host 180 rpm; feed 3 rpm) and made into testable standard samples by an injection molding machine. Finally, the obtained composites with different inorganic fillers were coded as A1~A9.

### 2.3. Characterization

A servo-controlled tensile testing machine (Gt-ai7000-l10, Taiwan High-speed Railway Testing Instrument Co., Ltd., Taiwan, China) was used to test the mechanical properties of the samples. The tensile and binding test was used according to GB 1040 and GB 1042, respectively. The impact test machine (Jbs-3002, Jinan Liangong Testing Technology Co., Ltd., China) was used to test the impact strength of the sample. The cantilever impact test adopted the provisions of GB/T 1843-2008, and the impact pendulum energy was 1 J. Ten specimens were needed for each test.

The microstructure of the fracture surface of the different PLA/PBS composites was observed by scanning electron microscope (Nano SEM 200, Fei Company, Hillsboro, OR, USA) at an accelerating voltage of 5 kV. Before SEM observation, the fracture surface of the specimens was sprayed with gold.

The Vicat softening point (VST) of the sample was tested with a computerized thermal deformation temperature tester (CRS-VST, Suzhou Yanuotianxia Instrument Co., Ltd., China). The measurement was carried out in a silicone oil bath with a heating rate of 2 °C/min. The average value of the sample was calculated by three repeated tests. To measure VST, a constant load of 10 N was applied to the rectangular specimen by a flat head needle with a cross-sectional area of 1 mm^2^, and the VST was determined when the needle perforated the specimen to a depth of 1 mm.

TG test was carried out using a thermogravimetric analyzer (Diamond TG-DTA/Spectrum GX, PerkinElmer, Japan/UK). Next, 5~10 g samples were taken and the temperature was increased from 40 °C to 600 °C in a nitrogen environment at a rate of 10 °C/min.

A low-temperature differential scanning calorimeter (Q1000, Ta company, USA) was used for the DSC test. Taking 5~10 g samples, the temperature was increased from 40 °C to 200 °C in a nitrogen environment at the speed of 10 °C/min, then the temperature was decreased from 200 °C to 40 °C at the speed of 10 °C/min, and finally the temperature was increased from 40 °C to 200 °C at the speed of 10 °C/min.

## 3. Results and Discussion

### 3.1. Mechanical Properties

#### 3.1.1. Tensile Properties

**Figure 1:**
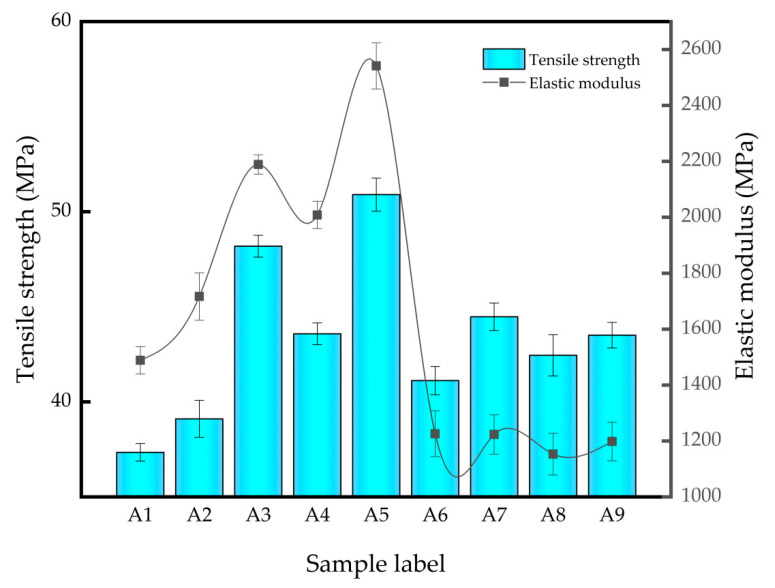
Tensile strength and elastic modulus of different PLA/PBS composites.

The tensile strength and elastic modulus of the different inorganic filler reinforced PLA/PBS composites were measured by tensile experiments, as shown in Figure 1. From Figure 1 it can be seen that the addition of GF, GF/SiO_2_ and GF/PAC make the PLA/PBS composites have a significantly higher tensile strength and elastic modulus compared to the pristine PLA/PBS composite, which was due to the high tensile properties of the inorganic filler. For the pristine PLA/PBS composite, the tensile strength and elastic modulus were 37.35 MPa and 1488.5 MPa, respectively. With the increasement in the glass fiber content, the tensile strength and elastic modulus both increased. When the glass fiber content was 10 wt%, the tensile strength and elastic modulus increased by 23.72% and 47.06%, respectively, which was due to the high tensile properties of the glass fiber itself. When using GF/SiO_2_ as the combination filler to synergistically reinforce the PLA/PBS composite, it can be seen that the tensile properties of the PLA/PBS composites showed an enhancement trend with the increase in the proportion of SiO_2_, and the tensile properties of A5 was the highest of the different PLA/PBS composites. Compared to the pristine PLA/PBS composite, the tensile strength and modulus of A5 increased by 36.28% and 70.74%, respectively. When using GF/PAC as the combination filler to synergistically reinforce the PLA/PBS composite, it can be seen that with the increase in PAC content, the A7 obtained the highest tensile strength and modulus among A6~A8, which was due to the good dispersion state of the PAC in the PLA/PBS matrix. For A8 composites, the tensile properties were slightly lower than A7, which could be ascribed to the poor dispersion of the high-content PAC. Furthermore, at the same glass fiber content, the A9 exhibited a relatively higher tensile strength compared to A6, which was due to the relatively longer glass fiber (12 mm) in the A9 composite that was in favor of the increment in the composites’ tensile strength and modulus.

#### 3.1.2. Flexural Properties

**Figure 2:**
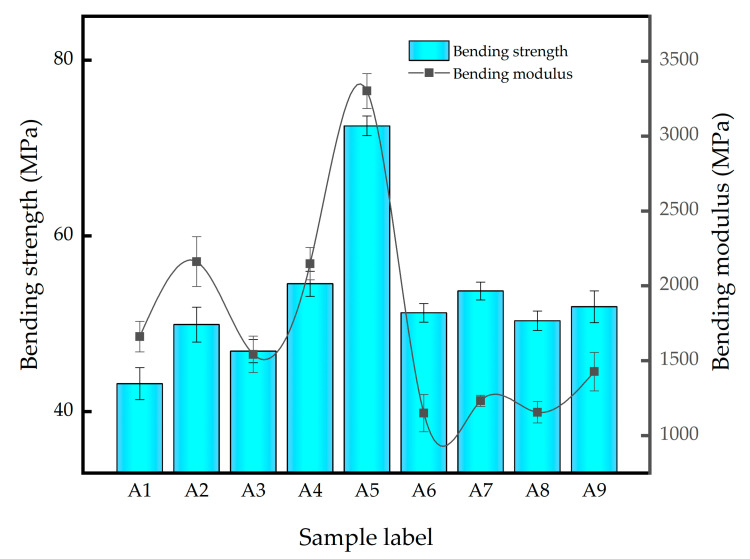
Flexural strength and modulus of different PLA/PBS composites.

The bending properties of different inorganic fillers reinforced PLA/PBS composites were tested and the results were shown in Figure 2. It can be seen that compared with the pristine A1 composite, the bending properties of GF, GF/SiO_2_ and GF/PAC reinforced PLA/PBS composites were all improved, and their reinforcing effect was the same as that on the tensile properties.

Among them, for the GF/SiO_2_ combined reinforced PLA/PBS composites, the bending properties show a positive correlation with the increase in SiO_2_, and the addition of 10%GF/1%SiO_2_ has the best reinforcing effect; that is, the bending performance of the A5 composite was the highest. Compared with the matrix composite A1, the bending strength and modulus of A5 increased by 67.95% and 66.61%, respectively. When using GF/PAC as the combination filler to synergistically reinforce the PLA/PBS composite, it can be seen that A7 obtained the highest flexural strength and modulus among A6~A8, which was due to the good dispersion state of the PAC in the PLA/PBS matrix. For A8 composites, the flexural properties were slightly lower than A7, which could be ascribed to the poor dispersion of the high-content PAC. Furthermore, when using the relatively longer glass fiber (12 mm), the A9 exhibited relatively higher flexural properties compared with A6, the reason being that the relatively longer glass fiber was in favor of the increment in the composites’ bending properties.

#### 3.1.3. Impact Properties

**Figure 3:**
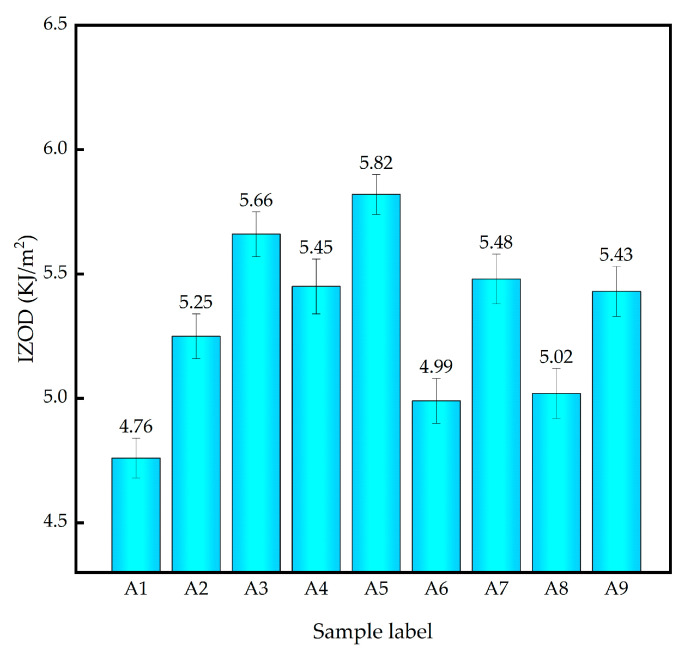
Izod impact strength of different PLA/PBS composites.

The Izod impact strength of different inorganic fillers reinforced PLA/PBS composites were tested and the results were shown in Figure 3. It can be seen that the impact strength of the modified composites was significantly improved compared with that of the pristine PLA/PBS composites, indicating that GF, GF/SiO_2_ and GF/PAC all possess excellent impact strength reinforcement functions. However, there are still some differences in impact strength between the modified composites. Among them, the 10%GF/1%PAC reinforced PLA/PBS composite (A5) had the best improvement effect. Compared with the pristine A1 composite, the impact strength was increased by 135.68%.

**Figure 4:**
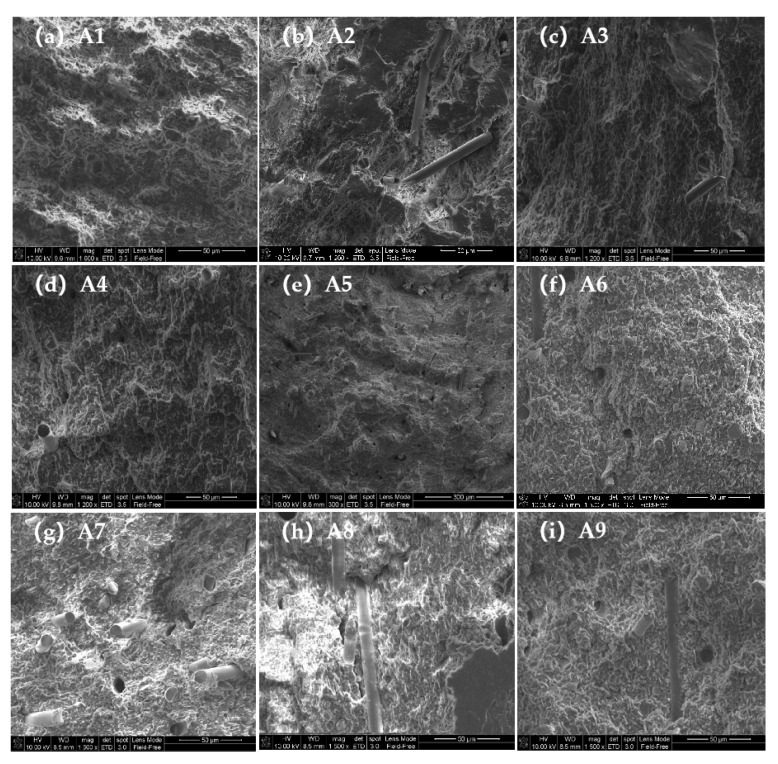
SEM images of different PLA/PBS sample’s impact fracture surface morphology: (**a**) A1; (**b**) A2; (**c**) A3; (**d**) A4; (**e**) A5; (**f**) A6; (**g**) A7; (**h**) A8; (**i**) A9.

The reinforcement of the Izod impact strength can be explained by Figure 4. Figure 4 shows the SEM images of different PLA/PBS samples’ impact fracture surface morphology. As shown in Figure 4, the fracture surface of the PLA/PBS matrix (A1) is relatively smooth, which indicates the relatively low toughness. When the PLA/PBS matrix was reinforced by glass fiber, some fiber fractures or holes can be seen left on the sample’s impact fracture surface; the fiber fracture and pulling out of the polymer matrix will consume a significant amount of impact energy when suffering impact damage. Additionally, the higher the glass fiber content, the higher the Izod impact strength; therefore, the A3 sample has a relatively higher Izod impact strength compared with A1 and A2. When the PLA/PBS matrix was reinforced by GF/SiO_2_, with the increase in SiO_2_ content, the fracture surface of the composite becomes coarser and coarser, which explains why the impact resistance of the composite becomes stronger. When the PLA/PBS matrix was reinforced by GF/PAC, there were still many holes and broken fibers in the fracture surface, implying improved toughness compared with the pristine PLA/PBS composite. However, the relatively smooth fracture surface compared with GF and GF/SiO_2_ reinforced PLA/PBS composites indicates a relatively weak impact strength. The reason can be ascribed to the weak compatibility between PAC and the polymer matrix.

### 3.2. Thermal Performance Analysis

**Figure 5:**
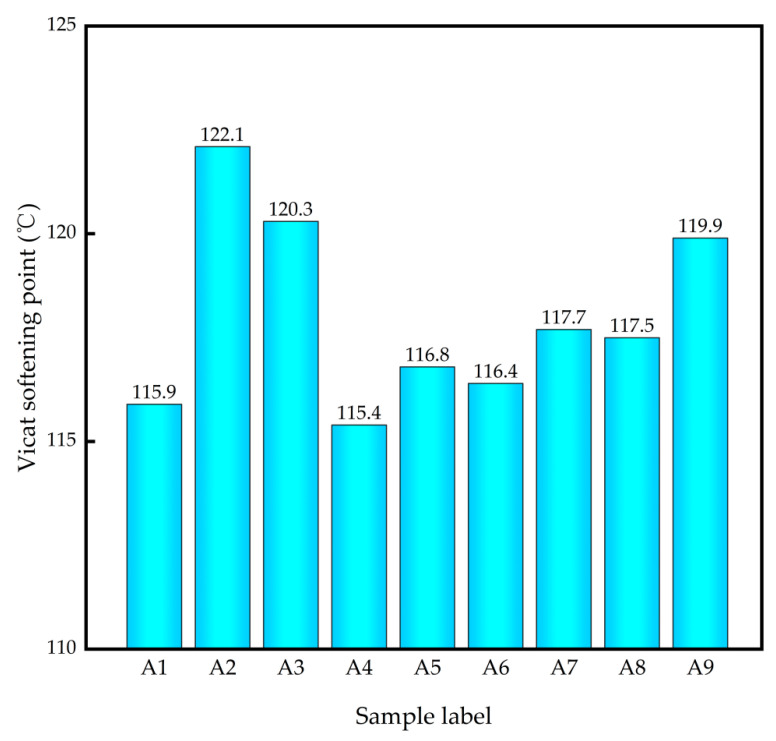
Vicat softening point of different PLA/PBS composites.

Figure 5 shows the Vicat softening point of different PLA/PBS composites. It can be seen that the Vicat softening point of the modified composite apparently improved, and the Vicat softening point of A2 was the highest (122.1 °C). For GF reinforced PLA/PBS composites, with the increase in GF content, the Vicat softening point of the modified composites increased at first and then decreased, reaching the highest value when the GF content was 5wt%, which is 5.3% higher than that of the pristine PLA/PBS composite. Although SiO_2_ and PAC can also improve the Vicat softening point of pure PLA/PBS matrix, the effect is not as good as GF, and there is no obvious relationship between the SiO_2_ or PAC content and the PLA/PBS composites’ Vicat softening point.

### 3.3. TG Analysis

**Figure 6:**
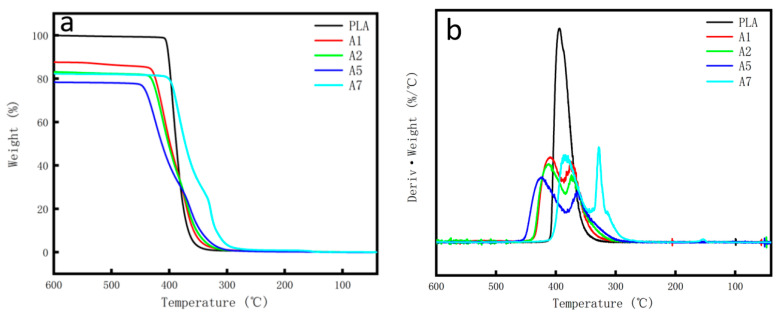
Thermogravimetric analysis of different PLA/PBS composites: (**a**) TG curves and (**b**) DTG curves.

It can be seen from Figure 6a that the thermal decomposition of pure PLA is completed in one step, while the thermal decomposition of composites is completed in multiple steps. According to the data in Table 2, T_s_, T_5%_, T_10%_ and T_50%_ represent the decomposition temperature when the initial weight loss rate of the material is 0%, 5%, 10% and 50%, respectively. The initial decomposition temperature of pure PLA is about 327.67 °C, which is higher than that of modified composites. However, it can be seen that the overall thermal decomposition rate of pure PLA is the fastest, and when the weight loss rate is 50% the thermal decomposition temperature is about 388.0 °C, which was relatively lower compared with the GF, GF/SiO_2_ and GF/PAC reinforced PLA/PBS composites. Moreover, it can be seen from Figure 6b that the thermal stability of different composites is A5 > A2 > A1 > PLA. The thermal decomposition process of the GF/SiO_2_ reinforced PLA/PBS composite is the slowest, and the thermal decomposition temperature at a 50% weight loss rate is about 412.83 °C, which is higher than the other modified composites, indicating that the GF/SiO_2_ reinforced PLA/PBS composite has better thermal stability.

### 3.4. DSC Analysis

**Figure 7:**
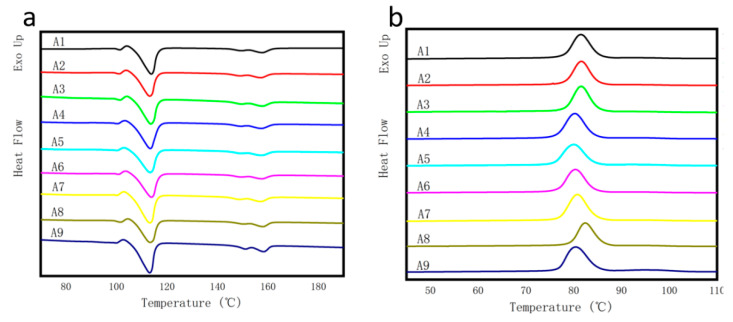
DSC analysis of different PLA/PBS composites: (**a**) melting curves and (**b**) crystallization curves.

The DSC analysis of the composite was further carried out. The melting curve and crystallization curve of the composite were shown in Figure 7a,b, respectively. Additionally, the crystallization temperature ($T_{c}$), crystallization enthalpy ($\Delta H_{c}$), melting temperature ($T_{m}$), melting enthalpy ($\Delta H_{m}$), cold crystallization temperature ($T_{\mathrm{cc}}$) and cold crystallization enthalpy ($\Delta H_{\mathrm{cc}}$) of the different composites were shown in Table 3. The crystallinity (χ) of the composites was calculated according to Formula (1) [40],

(1) $$\chi =\frac{\Delta H_{m}-\Delta H_{\mathrm{cc}}}{\Delta H_{m}^{0}}\times 100\%$$

where $\Delta H_{m}^{0}$ is the melting enthalpy of pure PLA, calculated as 93.6 J/g, $\Delta H_{m}$ is the melting enthalpy of the composite, $\Delta H_{\mathrm{cc}}$ is the cold crystallization enthalpy of the composite [40].

It can be seen from Table 3 that the crystallinity of the A1 composites is 27.36%, and the crystallinity of the modified composites is generally higher than that of the matrix composites. At the same time, the crystallinity of the GF reinforced PLA/PBS composite A2 is 32.35%, which is about 18% higher than that of the matrix composites A1, indicating that the addition of GF has a certain nucleation effect and increases the crystallinity of composites; the crystallinity is closely related to the heat resistance of the composites. Generally, the better the crystallinity of the composites, the higher the heat resistance [6], which also explains the high Vicat softening point of the A2. At the same time, the crystallinity of A4 and A5 is slightly lower than that of matrix composites. It is speculated that SiO_2_ modification has a certain interference on the movement of PLA molecular chain, the movement ability of molecular chain decreases, and the long chain affects the regularity of the composites, so the crystallinity decreases slightly.

It can be seen from Figure 7a that the cold crystallization peak of different PLA/PBS composites is concentrated at about 100 °C, and with the addition of GF, GF/SiO_2_ and GF/PAC, the melting peak of the composites gradually moves to the low-temperature region, and the secondary melting peak at 160 °C appears in the curve with the increase in temperature, which could be ascribed to the high melting point of PLA, which was in agreement with the peak at 160 °C of the DSC peaks in Figure 6b. It can be seen from Figure 7b that with the addition of GF and SiO_2_, the crystallization peak of the composites shifts to the low-temperature region, but with the addition of PAC, the crystallization peak of the composite shifts to the high-temperature region, and the degree of shift is different.

## 4. Conclusions

In this paper, to enhance the toughness and heat resistance properties of the PLA/PBS composites, the PLA/PBS polymer matrix was modified by different GF, GF/SiO_2_, and GF/PAC fillers. Additionally, the effect of filler type, filler content and their synergistic effect on the mechanical and thermal properties of the PLA/PBS composites was researched. It was found that the addition of GF, GF/SiO_2_ and GF/PAC make the PLA/PBS composites appear significantly higher regarding their tensile, flexural and impact properties compared with the pristine PLA/PBS composite. Among the different inorganic fillers, the 10%GF/1%SiO_2_ fillers showed the best mechanical properties enhancement function. Additionally, the tensile strength, elastic modulus, flexural strength, flexural modulus and Izod impact strength improved by 36.28%, 70.74%, 67.95%, 66.61% and 135.68%, respectively. Although the A2 owns the highest Vicat softening point (122.1 °C) and crystallinity (32.35%) due to the addition of GF, which has a certain nucleation effect and is in favor of the increment in the composites’ crystallinity, the A5 composites still had relatively higher thermal stability: when the weight loss rate was 50% the thermal decomposition temperature was the highest at 412.83 °C, and its Vicat softening point was up to 116.8 °C. In a word, the 10%GF/1%SiO_2_ reinforced PLA/PBS composites A5 exhibit excellent mechanical and thermal properties, which shows their potential industrial application prospects.

## Figures and Tables

**Table 1:** Material proportioning.

Sample	Talc/wt%	ADR4468/wt%	TBC/wt%	Eru/wt%	GF/wt%	SiO_2_/wt%	PAC/wt%
A1	14.8	1	0.37	0.1	/	/	/
A2	14.8	1	0.37	0.1	5 (3 mm)	/	/
A3	14.8	1	0.37	0.1	10 (3 mm)	/	/
A4	14.8	1	0.37	0.1	5 (3 mm)	0.5	/
A5	14.8	1	0.37	0.1	10 (3 mm)	1	/
A6	14.8	1	0.37	0.1	5 (3 mm)	/	0.5
A7	14.8	1	0.37	0.1	5 (3 mm)	/	1
A8	14.8	1	0.37	0.1	5 (3 mm)	/	3
A9	14.8	1	0.37	0.1	5 (12 mm)	/	0.5

**Table 2:** Thermal decomposition temperature of PLA and composites.

Sample	T_s_/°C	T_5%_/°C	T_10%_/°C	T_50%_/°C
PLA	327.67	358.67	367.00	388.00
A1	294.17	345.67	357.83	398.83
A2	303.67	338.50	352.67	401.33
A5	298.17	330.33	346.17	412.83
A7	250.35	306.43	318.66	372.26

**Table 3:** DSC characteristic parameters of different composites.

Sample	T_CC_/°C	ΔH_CC_/J·g^−1^	T_1m_/°C	ΔH_1m_/J·g^−1^	T_2m_/°C	ΔH_2m_/J·g^−1^	T_C_/°C	ΔH_C_/J·g^−1^	χ/%
A1	102.45	1.048	113.79	26.66	157.79	6.338	81.53	29.22	27.36
A2	101.07	1.403	113.39	30.75	157.52	7.236	81.58	31.52	32.35
A3	102.72	1.339	113.58	30.23	157.43	7.923	81.61	32.67	30.87
A4	102.29	1.105	113.59	25.69	157.56	6.471	80.31	28.45	26.26
A5	101.07	1.133	113.59	26.59	157.62	6.757	79.98	27.67	27.20
A6	101.76	1.275	113.98	26.79	157.74	5.865	80.30	28.85	27.26
A7	102.39	1.031	113.29	28.02	157.01	6.964	80.84	30.89	28.83
A8	101.44	1.065	114.09	27.57	157.80	6.504	82.49	29.26	28.32
A9	100.63	1.242	113.15	29.34	158.29	7.799	80.50	29.19	30.02
